# A Guideline for Contextual Adaptation of Community-Based Health Interventions

**DOI:** 10.3390/ijerph19105790

**Published:** 2022-05-10

**Authors:** Zinzi E. Pardoel, Sijmen A. Reijneveld, Maarten J. Postma, Robert Lensink, Jaap A. R. Koot, Khin Hnin Swe, Manh Van Nguyen, Eti Poncorini Pamungkasari, Lotte Tenkink, Johanna P. M. Vervoort, Johanna A. Landsman

**Affiliations:** 1 Department of Health Sciences, University Medical Center Groningen, University of Groningen, Hanzeplein 1, 9700 RB Groningen, The Netherlands; s.a.reijneveld@umcg.nl (S.A.R.); m.j.postma@rug.nl (M.J.P.); j.a.r.koot@umcg.nl (J.A.R.K.); lottetenkink@gmail.com (L.T.); j.p.m.vervoort@umcg.nl (J.P.M.V.); j.a.landsman@umcg.nl (J.A.L.); 2 Department of Pharmacology and Therapy, Faculty of Medicine, Universitas Airlangga, Surabaya 60115, Indonesia; 3 Centre of Excellence in Higher Education for Pharmaceutical Care Innovation, Universitas Padjadjaran, Bandung 45363, Indonesia; 4 Department of Economics, Econometrics & Finance, Faculty of Economics and Business, University of Groningen, 9747 AE Groningen, The Netherlands; b.w.lensink@rug.nl; 5 HelpAge International, Yangon 11081, Myanmar; swe.khinhnin@helpagemyanmar.org; 6 HelpAge International, Hanoi 1000, Vietnam; manhnv@helpagevn.org; 7 Department of Public Health, Faculty of Medicine, Universitas Sebelas Maret, Surakarta 57126, Indonesia; etiponco@staff.uns.ac.id

**Keywords:** cultural context, guideline, adaptation, participatory action research, co-creation, Positive Health, community-based health interventions

## Abstract

In Southeast Asia, community-based health interventions (CBHIs) are often used to target non-communicable diseases (NCDs). CBHIs that are tailored to sociocultural aspects of health and well-being: local language, religion, customs, traditions, individual preferences, needs, values, and interests, may promote health more effectively than when no attention is paid to these aspects. In this study, we aimed to develop a guideline for the contextual adaption of CBHIs. We developed the guideline in two stages: first, a checklist for contextual and cultural adaptation; and second, a guideline for adaptation. We performed participatory action research, and used the ‘Appraisal of Guidelines for Research & Evaluation (AGREE) II’ tool as methodological basis to develop the guideline. We conducted a narrative literature review, using a conceptual framework based on the six dimensions of ‘Positive Health’ and its determining contexts to theoretically underpin a checklist. we pilot tested a draft version of the guideline and included a total of 29 stakeholders in five informal meetings, two stakeholder meetings, and an expert review meeting. This yielded a guideline, addressing three phases: the preparation phase, the assessment phase, and the adoption phase, with integrated checklists comprising 34 cultural and contextual aspects for the adaption of CBHIs based on general health directives or health models. The guideline provides insight into how CBHIs can be tailored to the health perspectives of community members, and into the context in which the intervention is implemented. This tool can help to effect behavioral change, and improve the prevention and management of NCDs.

## 1. Introduction

In Southeast Asia, community-based health interventions (CBHIs) are often implemented to target non-communicable diseases (NCDs) [1,2,3]. There is some indication that CBHIs—that are tailored to sociocultural aspects of health and well-being: local language, religion, customs, traditions, individual preferences, needs, values, and interests—may promote health more effectively than when no attention is paid to these aspects [4,5,6]. CBHIs are complex social processes involving multiple components such as screening, physical exercise and education, and are aimed at preventing illness and unhealthy behavior and promoting the well-being of various population groups [1]. These CBHIs address health risk behaviors that are major determinants of NCDs, such as tobacco smoking, alcohol, unhealthy diet, and physical inactivity. CBHIs aim to improve these behaviors by using primary health care to increase people’s knowledge about health, solidarity, self-reliance, social support, and synergy [7]. Health behaviors are determined to a significant degree by health perception, which is highly context-dependent [8], i.e., formed in the demographic, healthcare, cultural and social contexts to which an individual belongs [9,10]. For this reason, CBHIs must be culturally adapted to the contexts of the participants to effectively bring about behavioral change and improve the prevention and management of NCDs [11].

**Figure 1:**
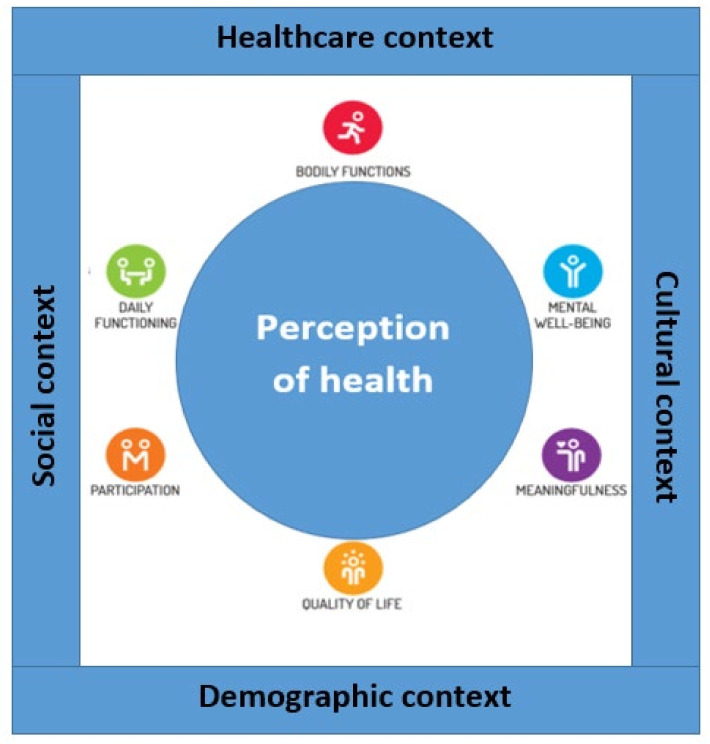
Conceptual framework: the six dimensions of health perception and its determining contexts. Based on Huber’s ‘Concept of Positive Health’ [14,15].

In 1948, the World Health Organization (WHO) defined health as “a state of complete physical, mental and social well-being and not merely the absence of disease or infirmity” [12]. Although this aspirational definition of health is accepted worldwide, it does not always align with health perceptions. Huber’s concept of ‘Positive Health’ [13,14] provides a theoretical framework to accommodate the impact of context on peoples’ individual health perception, making it a helpful guide for the adaptation of such interventions. The concept consists of six major dimensions: *bodily functions*, *mental well-being*, *meaningfulness*, *participation*, *daily functioning*, and *quality of life*. According to this concept, health is established in and influenced by peoples’ health perception, which is in turn associated with their cultural, social, healthcare, and demographical environments (see Figure 1). Therefore, incorporating the dimensions of ‘Positive Health’ can help to adapt CBHIs to the context in which they are to be applied, thus, enhancing the promotion, prevention, and management of health. 

CBHIs are commonly developed based on validated directives, models, and approaches, such as the WHO‘s Integrated Care for Older People (ICOPE)—community-level interventions for managing the decline in intrinsic capacity [16]. 

To date, several studies address the guidance for the adaptation of existing interventions to context. First, the ADAPT guideline [17] offers a framework to help researchers, policy and practice stakeholders, funders, and journal editors in undertaking and assessing the adaptation of interventions for a new context, and reporting these transparently. Second, the report of Graig and colleagues provides guidance on how context should be taken into account, from priority setting and intervention development to the design and conduct of evaluations and reporting, synthesis, and knowledge exchange [18]. These two guidelines provide extensive and in-depth information for the adaptation and reporting process of (health) interventions. However, to our knowledge, there are no concrete, practical, scientifically based guidelines available to adapt CBHIs to a specific context, even though this is considered to be an important aspect of most guidelines [19]. This study, therefore, aimed to develop such a concrete and practical guideline, and was part of the EU-H2020 funded project, “Scaling up Non-communicable disease interventions in South East Asia” (SUNI SEA), taking place in Myanmar, Indonesia, and Vietnam, and coordinated from The Netherlands.

## 2. Materials and Methods

### 2.1. Design

**Figure 2:**
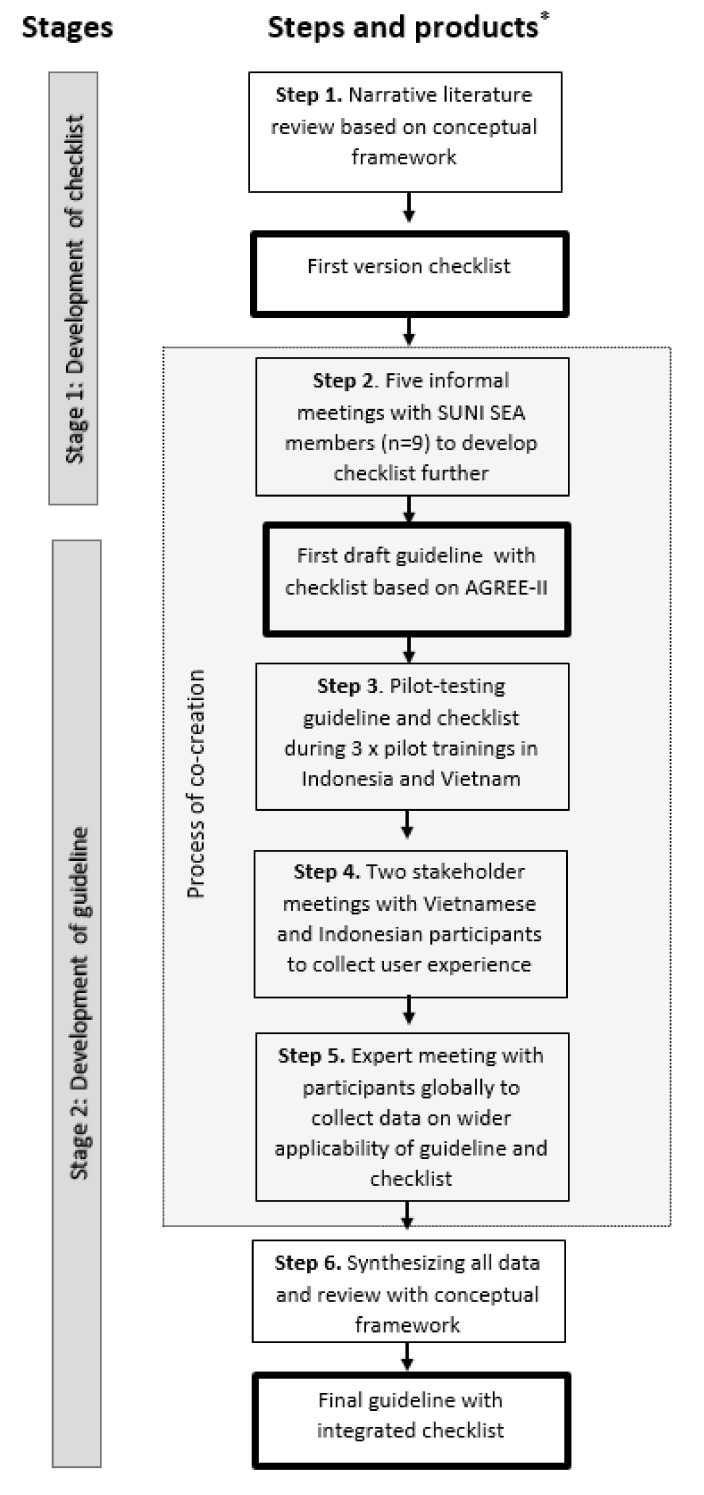
Design of the study structured in stages, steps, and products. * Boxes with bold outline denote products created in the process.

We developed the guideline in two stages with six steps: first, a checklist for contextual and cultural adaptation; and second, a guideline for adaptation, which includes the checklist (see Figure 2). The conceptual framework of ‘Positive Health’ and its determining contexts formed the theoretical basis for our narrative literature review and for the inclusion of contextual and cultural aspects in the guideline. We derived the steps per stage using ‘Appraisal of Guidelines for Research & Evaluation (AGREE) II’ [20], and step-by-step assessed the issues raised by this instrument. The purpose of AGREE-II is to provide a framework to assess and achieve quality, and a methodological strategy for the development of guidelines. We then used a participatory action research (PAR) [21] approach to shape co-creation [22]: collaborative knowledge generation by academics working jointly with other stakeholders. 

### 2.2. Sample and Procedure

We collected data from multiple sources, using a number of samples (see Table 1). In both stages, 29 stakeholders were involved; data were collected during two-hour meetings, held online via platforms WebEx and Microsoft Teams, and audio recorded. All data were transcribed verbatim and analyzed using qualitative content analysis of transcripts. 

The first stage, development of the checklist, involved two steps. In step 1, we conducted a narrative literature review, based on the concept ‘Positive Health’ and its determining contexts. The second step involved informal meetings. For the latter, we used a PAR approach [21], which has two distinguishing characteristics: participation of stakeholders as partners in the research process, and commitment to action for social change [23]. In every step of our action research, we reflected on previous steps and findings, using McIntyre’s ‘Recursive Process of PAR’ (2008) [21]. This iterative process includes six activities: questioning, reflecting, investigating, developing, implementing and refining. 

The second stage of the study involved four steps, resulting in the final guideline. In every step we followed the criteria of the AGREE-II [20], which can be applied in any disease area, targeting any step in the healthcare continuum, including health promotion, screening, or interventions. Because the AGREE-II was developed specifically for clinical guidelines, we adjusted the tool to make it more compatible for our research: we converted conditions or health issues into aspects of context. We then used the tool to guide the development of the guideline, covering all of its domains with several strategies. The AGREE-II consists of 23 key items within six domains. We covered the first domain, scope and purpose, by adding to the guideline a description of the objectives, the questions, and the target group. For the second domain, stakeholder involvement, we held meetings with various stakeholders. For the third domain, rigor of development, we conducted mixed-method research; for the fourth, clarity of presentation, we consulted with various stakeholders and experts to assure transparency, readability, and clarity. For the fifth domain, applicability, we conducted pilot testing and multiple evaluations of the guideline; this resulted in an extensive description of applicability, as well as possible barriers and facilitators. Finally, for the sixth domain, editorial independence, we recorded and addressed the issues of funding body and competing interests.

In the last step, we synthesized, reviewed and reflected upon all data using the conceptual framework. This led to the finalized guideline with the checklist.

**Table 1:** Overview of data samples and characteristics.

Data Sample	Narrative Literature Review	Informal Meetings	Stakeholder Meetings	Expert Meeting
Characteristics				
Phase	Phase 1 development of checklists	Phase 1 development of checklists	Phase 2 development of the guideline. After pilot testing the guideline.	Phase 2 after stakeholder meetings
Number of articles/persons	13	9	15	5
Sampling method	Databases: PubMed, Google, Psych info and snowball method	Members of SUNISEA consortium	Convenience sampling [24], starting with stakeholders involved in the pilot trainings	Convenience sampling the expert pool of HelpAge International
Involvement with CHBIs	-	Development, research and/or implementation of CBHIs	Observers in pilot trainings or involved in the research or implementation of interventions	Country directors of NGOs involved in CBHIs
Gender	-	3 males/6 females	5 males/10 females	2 males/3 females
Years/Age range	1993–2020	28–63 years	2 –53 years	32–55 years
Countries	Asian countries	2 Indonesia, 1 Myanmar, 2 Vietnam, 4 Netherlands	10 Indonesia 5 Vietnam	1 Moldova, 1 The Philippines, 1 Sri Lanka, 1 Cambodia, 1 Vietnam
Period	April–May 2020	May–June 2020	January–February 2021	September 2021

The two stages of our study included the following activities:

### 2.3. Stage 1: Development of a Checklist for Contextual Adaptation of the CBHIs

In the first stage, we developed a checklist. In the first step, we conducted a narrative literature review [25] in April and May to establish focus for the checklist. In this review, articles were included that covered contextual and cultural aspects of Asia within the six dimensions of ‘Positive Health’. Moreover, only the literature in English was included. Articles were excluded when they did not cover Asian countries nor cultural and contextual aspects within the six domains. We used the databases PubMed, Google, and Psych info, combining different key words such as “Positive Health”, “Health Perception”, “Cultural aspects”, “Context”, and “Interventions”. To find the literature, we used the snowball method, using the bibliography or footnotes of a paper to identify additional papers [26]. Based on the findings of this review, we developed a first draft of the checklist.

The second step involved consultations via informal meetings with stakeholders (n = 9) who were working on the development, research, and/or implementation of the CBHIs. The draft of the checklist was disseminated in the SUNI-SEA consortium in May and June 2021. Based on the feedback in the meetings, we began the development of the guideline. 

### 2.4. Stage 2: Development of a Guideline for Application of the Checklist

In the second stage, we developed a guideline using steps 3, 4, 5, and 6. In the third step, we pilot tested the draft version of the guideline, including the checklist, during the development of materials, and implementation of a community-based training (CBT) for organizing and giving the CBHIs. In December 2020 and January 2021 in Indonesia and Vietnam, during the implementation of the CBT, independent observers used the checklist and guideline to examine the cultural and contextual aspects of the training. In Vietnam, the draft version was pilot tested during two training sessions, with 35 members and one observer per session. In Indonesia, the draft version was pilot tested once within a group of 20 members and one observer. Observers filled in evaluation forms regarding their experiences with the implementation of the checklist and guideline. In the fourth step, in January and February 2021, we organized stakeholder meetings. The topic list for the stakeholder meeting was based on the five areas of focus for feasibility studies by Bowen and colleagues [27], namely: (1) practicality (e.g., “To what extent can guideline be carried out with intended participants using existing means, resources, and circumstances?”); (2) adaption (e.g., “To what extent does the guideline perform when changes are made for a new format or with a different population?”); (3) acceptability (e.g., “To what extent is the guideline judged as suitable, satisfying, or attractive to program deliverers or program recipients?”); (4) implementation (e.g., “To what extent can the guideline be successfully delivered to intended participants in some defined, but not fully controlled, context?”); and (5) demand (e.g., To what extent is the guideline likely to be used). In the fifth step, in September 2021, we held an expert review meeting. In the expert meeting, the topics were based on the five focus areas regarding feasibility studies as proposed by Bowen and on the results of the stakeholder meetings. The findings of the stakeholder and expert meetings were categorized under facilitators and barriers based on the revisions made to the guideline and checklist. As a sixth step, we synthesized, reviewed, and reflected upon all data within the conceptual framework. This step concluded the co-creation phase, and the researchers finalized the guideline with the checklist. 

## 3. Results

### 3.1. Stage 1: Development of the Checklist

The findings of the first step, the literature review, are presented in Appendix A, Table A1, illustrating the dimensions related to cultural or contextual aspects. We found 13 articles, including reviews, explorative studies and qualitative studies, covering Asian contextual and cultural aspects within the dimensions of ‘Positive Health’. The perception of ‘bodily functions’ in Southeast Asia is comprehensive; physical health is conceptualized as the harmony and unity of mind, body, and soul [28]. As Ravindran et al. (2012) explain, “upset in body balance is the common way to look at disease; it refers to the belief that a healthy body is in a state of balance. When the body gets out of balance, illness results” [28]. A number of factors can disturb this balance, such as certain foods, medications, herbs, or strong emotions. Therefore, what you eat or emotionally feel can directly influence your organs and your bodily functioning [29]. A contextual aspect within the ‘mental well-being’ dimension is the stigmatization of mental illness. In Southeast Asia, emotional expression is commonly considered to be personal weakness, and can contribute to being stigmatized with mental illness [30]. Such stigma is also grounded in the rigidity of restraint societies in Southeast Asia, where the predominantly practiced Buddhism looks upon mental illness as suffering caused by one’s own past misdeeds [31]. For the ‘meaningfulness’ dimension, we found that in different cultural societies, factors that give life meaning are often found in spiritual and religious beliefs [32]. Most countries in Southeast Asia are multicultural, with many minority groups, resulting in a variety of religions [30]; the most commonly practiced religion is Buddhism, whose basic principles are often familiar to people of other religions. The ‘participation’ dimension depends on a balance between opportunities and limitations [14]. Associated with this balance is the ability to participate and being actively involved in ordinary family and community activities [33]. Southeast Asian countries often have a high intergenerational co-residence, where children take care of their parents [30]. The Southeast Asian elderly participate mainly by giving advice to family and community members. Their accumulated life wisdom and spiritual capacities make this advice highly appreciated [33]. Further, contextual aspects for ‘daily functioning’ include current or past work-related activities and availability of/barriers to healthy food. A study by Nilsson et al. (2005) indicated that in Southeast Asia, being functional in daily life is ‘having the strength and physical ability to work’ [33]. Another determiner of daily functioning is having good health, which in turn can be related to food [33]. According to Huber, one aspect of the ‘quality of life’ dimension is happiness. Uschida et al. (2004) indicated that the cultural meaning of happiness in Southeast Asia is defined mainly in terms of interpersonal connectedness [34].

In the second step, we collected feedback from several stakeholders (n = 9) in informal meetings. This yielded additional new aspects of context: adult friendly methods, cultural and individual exercise options, and the role of a trainer/implementer (e.g., role model for a healthy lifestyle). However, as some topics turned out to be ambiguous and difficult to understand, we added an appendix with definitions and meanings to the guideline. A further important finding from the meetings was the need for a guideline with practical information on how to apply the checklist. We, therefore, jointly agreed to include instructions for the use of the checklist in our guideline. 

In the second stage, we developed a guideline.

### 3.2. Stage 2: Development of the Guideline

The draft of the guideline included basic practical information on how to apply the checklist. In the third step, we pilot tested the guideline. The guideline, including the checklist, was perceived as a helpful tool for addressing important contextual aspects during the development of training materials, and for monitoring the implementation of the training. In both countries, materials and trainings were adjusted based on the checklist. In Indonesia, information about traditional medicines and herbs (e.g., traditional therapy and alternative medicines) and the stigmatization of illnesses and mental health was added to the training. In Vietnam, the guideline was pilot tested in different areas (i.e., rural and urban), resulting in a different adaptation of the training to an urban or to a rural context, even though it was implemented in the same country. 

In the fourth step, we held stakeholder meetings to discuss the results of pilot testing the guideline (n = 15). We categorized the results under perceived facilitators of and barriers to the guideline and made appropriate revisions. Appendix B, Table A2, gives an overview of these results, including quotations that illustrate the findings. The main facilitator mentioned by the stakeholders was that the checklist and guideline were useful for adapting a (medical) message to the appropriate culture and context. Moreover, the stakeholders indicated that the guideline should be made adaptable to different contexts (e.g., countries, areas, groups), because several contextual aspects vary within different contexts. Based on this finding, we added an explanation—that cultural and contextual aspects differ per context, that some aspects are more important than others, and that some aspects can be irrelevant for certain contexts. The main barriers mentioned by the stakeholders were time allocation and translation, which we discussed further in the expert review meeting.

*“Some medical words were used in the materials that health practitioners use. However, not all community members know these words. Based on the checklist, we found this and revised this.”.*

(Stakeholder from Indonesia)

In the fifth step, several international experts (n = 5) discussed the guideline with the checklist, providing an in-depth review of the guideline. Their views and ideas converged with those in the stakeholder meetings, as shown in Appendix B. The experts found the guideline to be innovative and essential for developing the CBHIs. One of their main recommendations was to involve stakeholders: community members, representatives, government and funding parties. Involvement of stakeholders can reduce the barriers of translation and time. However, effective involvement of stakeholders requires appropriate allocation of budget, and also time. A new barrier mentioned by the experts was implementers not always being role models for the participants of the CBHIs and they recommended to carefully reconsider the word ‘role model’.

*“It is too sensitive that an implementer or a trainer should be a role model for a healthy lifestyle. Someone can still deliver the message of healthy lifestyle and be overweight.”.*

(Expert from Cambodia)

As the sixth step, we synthesized all data into the final guideline with the checklist. The final guideline is included in the Supplementary Material of this article. We expanded the final guideline to include background information as well as an introduction explaining the concept of ‘Positive Health’ and the importance of adjustment. Moreover, we added, as a recommendation, the process of cultural or contextual adaptation of CHBIs in three phases: the preparation phase, the assessment phase, and the adoption phase. These phases give guidance regarding the adaptation of materials and the implementation of CBHIs. Because the implementation of an intervention consists of more contextual aspects, such as the role of a trainer, compared to the adaptation of merely written intervention materials, the guideline includes two checklists: one for the materials and one for the implementation. The checklist for the implementation of an intervention is shown in Table 2. It includes 34 contextual and cultural aspects belonging to the topics: general aspects, the six dimensions of ‘Positive Health’, and the role of the implementer. Moreover, in the last step, we reflected upon the contextual aspects, using the conceptual framework; this showed that health perception is mostly influenced by aspects within the cultural and demographic context.

## 4. Discussion

We developed a guideline for the contextual adaption of CBHIs. We performed this in two stages, using seven steps derived from the AGREE-II tool. In the first stage, we used the conceptual framework of ‘Positive Health’ (based on determining contexts and health perception) to form the basis of a checklist for contextual adaptation. After a narrative literature review and informal meetings, we drafted our checklist. In the second stage, we developed a guideline (including the checklist), using a participatory action research approach involving pilot testing and multiple forms of co-creation with different stakeholders. 

This guideline is valuable for adapting existing or newly developed CBHIs to culture and context. To our knowledge, it is the first practical evidence-based guideline with a checklist to address the contextual adaptation of CBHIs with concrete examples and issues. From the literature, we know that a good fit between interventions and context requires careful adaptation [17]. The aim in developing the checklist was, thus, to create a tool to adapt CBHIs to context, which is necessary when implementing an existing CBHI in another area, country, or culture. According to Schloemer and Schröder-Bäck (2018), the transferability of existing interventions is complex, and a good fit between an intervention and the context is greatly affected by similarities and differences in the original and new contexts [35]. Transfer of interventions to other contexts has often been ineffective because such contextual aspects were ignored [17]. 

CBHIs are commonly developed using validated and evidence-based health directives or models from major international organizations, such as the World Health Organization or the Global Alliance for Chronic Diseases. Based on these generally validated health directives or models, the checklist and guideline developed as a result of our research has the potential to adapt CBHIs to the local context.

This guideline reflects the needs and knowledge of the involved stakeholders. In all steps of its development, we used a participatory action research (PAR) approach to shape co-creation, engaging community stakeholders as equal partners. Involving stakeholders is crucial in the process of contextual adaptation of interventions, as well as in the development of the final version of the checklist and guideline. This finding corresponds with other research on the implementation of interventions in a new context [36,37]. Stakeholder involvement has two overarching benefits. First, collaboration with stakeholders provides an awareness of context [38]; the engagement of various stakeholders led to the identification of contextual aspects that would have been missed if the checklist was based only on a literature review. When adapting a CBHI to a particular community, local stakeholders can help to harmonize it with the dynamics and structures of the community, incorporating its contextual aspects. Second, collaboration with stakeholders creates a feeling of ownership within the community involved [37,39]. Local ownership is especially important for CBHIs because it can enable the co-funding and sustainability of interventions [40]; this is often a challenge due to a lack of beneficiaries for community-based projects. The focus of PAR research is to make action possible; this is achieved through stakeholders collecting and analyzing data, and then determining together what action should follow [41]. 

Another important conclusion derived from our use of the PAR approach was that clear instructions were needed for optimal application of the checklist. The stakeholders, with their diverse levels of expertise and involvement in CBHIs, shared converging views and ideas for the construction of such instructions. As a result, this guideline can be more widely applied to other CBHIs and in other countries. Furthermore, as indicated in the literature, a guideline with a checklist for contextual adaptation of CBHIs can lead to more effective interventions to promote health [1,4,5,6], but not only health. All stakeholders indicated that the usefulness of this guideline extends beyond health-related community-based interventions.

Compared to similar guidelines, such as the ADAPT guideline [17] and the report of Graig and colleagues [18], we conclude that our guideline is a valuable addition to guidelines for the contextual and cultural adaptation of interventions. Our guideline is a practical and concrete guideline that can be assessed by different levels of involved people, e.g., intervention developers, implementers, observers, and/or trainers. Moreover, the guideline provides insight into the need for change in the already existing interventions. Using the checklists can be carried out periodically to check if changes are needed due to changing the context and/or culture. Compared to other guidelines, this can be carried out with little effort and time, making it a concrete and practical tool for the improvement as well as development of an intervention. 

We can conclude that our conceptual framework provides insight on how CBHIs can be adapted to the health perspectives of CBHI members. The concept of ‘Positive Health’ and its determining contexts guided the direction of our literature search and determined what we included in the checklist. Because ‘Positive Health’ is a broad concept: “the ability to adapt and self-manage” [14], based on a holistic view of aspects of life such as participation and daily functioning, we expect our guideline to be more generally applicable in community-based interventions, focused not only on health, but also on issues such as reducing natural disasters, preventing bullying among youth, and addressing gender violence.

### 4.1. Strengths and Limitations

A strength of this study is the use of various qualitative research methods to develop the guideline with the checklist. The use of multiple qualitative methods led to methodological triangulation, which provided increased validity and enhanced the understanding of contextual aspects [42]. The use of participatory action research led to generating collaborative knowledge, contributing to the scientific and practical base of the guideline. Finally, the process of co-creation is a further strength of this research: involving various stakeholders deepened and enriched the development of the guideline. 

A limitation of this study is that the guideline has been pilot tested only in Asian countries, and most of the involved stakeholders were from Asia. This may lessen its applicability to other continents. However, a number of involved stakeholders and an expert from continents other than Asia affirmed that the checklist and guideline were promising for application in their own country or continent. A final limitation is that the same stakeholders were involved in both the development and pilot testing of the guideline; this could have led to positive bias in the assessment of the pilot findings [43]. 

### 4.2. Implications

This guideline can be used for contextual adaptation of community-based interventions in Southeast Asia. As both Southeast Asian and non-Southeast Asian stakeholders have reviewed the guideline, we expect it to be potentially applicable in regions other than Southeast Asia. However, to confirm its applicability in other regions, the guideline should be further tested on interventions in other settings. 

The scientific conceptual framework, used as a basis for their development, imply that the checklist and guideline are likely to promote health more effectively when adapted optimally to context. The guideline was developed in a rigorous way, including multiple research methods and a diverse range of stakeholders. However, additional research is needed to assess their effectiveness and validity in routine practice.

This guideline has the potential to be a tool for more general contextual adaptation of interventions. Future research and practice should focus on its application beyond community-based health interventions. 

## 5. Conclusions

We have developed a guideline with a checklist for the contextual adaptation of CBHIs. To our knowledge, this is the first guideline to provide a practical and scientific base for contextual adaptation of both newly developed and already implemented CBHIs. Moreover, its guidance is based on general international health directives and models. 

## Figures and Tables

**Table 2:** Checklist for cultural and contextual adaptation of community-based health interventions (CBHIs).

Topic	Contextual/Cultural Aspects	Yes	No
1. General	1a. Gender differences		
	1b. Ability to read/write		
	1c. Age friendly methods, addressing differences between generations; if end-users were adults, adult learning methods were applied		
	1d. Digital inclusion/exclusion		
2. Bodily functions	2a. Perception of own body		
	2b. Physical fitness (cultural and individual exercise options) and/or somatic complaints		
	2c. Coping with stress and stigmatization of illnesses		
3. Mental well-being	3a. Perceptions regarding health: individual differences		
	3b. Local health traditions		
	3c. Cultural influences in diet		
	3d. Cultural influences on healthy living		
	3e. Myths and facts regarding health promotion		
	3f. Stigmatization of mental health, main issues		
	3g. Psychological stress, sources		
	3h. Feeling supported: role of peers, working together on health		
	3i. Feeling of belonging: social cohesion, part of community		
	3j. Availability of/barriers to informal resources: relatives/friends		
	3k. Access to resources: Barriers to access healthcare and medicines		
	3l. Barriers to access health information		
4. Meaningfulness	4a. Religious and spiritual beliefs		
5. Participation	5a. Family structure: role of elders, in-laws and siblings		
	5b. Being able to participate, and having a role in usual community activities		
	5c. Being able to participate and having a role in usual family activities (earning money, cooking and cleaning)		
6. Daily functioning	6a. Availability of/barriers to healthy food		
	6b. Current/past working life		
7. Quality of life	7a. Social network, role of social structures in health, e.g., governmental and non-governmental organisations		
8. Role of implementer	8a. Does implementer represent or have knowledge of healthy lifestyle?		
	8b. Is implementer a role model for the target group?		
	8c. Is implementer culturally and linguistically matched to target group?		
	8d. Are participants treated equally and inclusively by implementer?		
	8e. No stigma or discrimination by implementer? Inclusiveness, stimulation of participants to come with solutions for local issues?		
	8f. Does implementer take into account cultural diversity of participants?		
	8g. Does implementer take into account different levels of participant knowledge?		
	8h. Does intervention enhance self-efficacy of participants?		
9. Lessons learned or other remarks:			

## Data Availability

All raw data are available upon request.

## Supplementary Materials

The following supporting information can be downloaded at: https://www.mdpi.com/article/10.3390/ijerph19105790/s1, Guideline for contextual adaption of community-based interventions.

## Appendix A

**Table A1:** Listing of the aspects of culture and context found in the literature review as addressed in the checklist, per dimension of ‘Positive Health’.

Dimensions	Findings in the Literature	Aspects of Culture and Context Addressed in Checklist
Bodily functions	The perception of bodily functions in Southeast Asia is comprehensive; physical health is conceptualized as the harmony and unity of mind, body, and soul [28]. As Ravindran et al. (2012) explain, “upset in body balance is the common way to look at disease; it refers to the belief that a healthy body is in a state of balance. When the body gets out of balance, illness results” [28]. A number of factors can disturb this balance, such as certain foods, medications, herbs, or strong emotions. Therefore, what you eat or emotionally feel can directly influence your organs and your bodily functioning [28,29].	Perception of own body
		Physical fitness (cultural and individual exercise options) and/or somatic complaints
		Coping with stress and stigmatisation of illnesses
Mental well-being	In Southeast Asia, emotional expression is commonly considered to be personal weakness; this can contribute to stigmatization of mental illness [30]. This stigma is also grounded in the rigidity of restraint societies in Southeast Asia, in which the predominant practice of Buddhism considers mental illness to be suffering caused by one’s past misdeeds [31]. Moreover, in Southeast Asia, great numbers of people are exposed to extreme stressors due to humanitarian crises related mainly to natural disasters [44]. However, people with adaptive strategies for coping with extreme stressors are less likely to experience mental health problems [33]. Antonovsky (1984) described this as a ‘sense of coherence’ [32]. A sense of coherence is subject, among others, to the manageability and meaningfulness of a difficult situation. Manageability depends on the degree to which someone feels that resources, both formal (healthcare services) and informal (relatives and friends), are at their disposal. The second component, meaningfulness, is described below.	Perceptions of health: individual differences
		Local health traditions identified
		Cultural influences on diet identified
		Cultural influences on healthy living
		Myths and facts regarding health promotion
		Stigmatisation of mental health main issues identified
		Psychological stress sources identified
		Feeling supported: role of peers, working together on health
		Feeling of belonging: social cohesion, part of community
		Barriers to access health information
		Availability of/barriers to informal resources: relatives/friends
		Access to resources: barriers to access healthcare and medicines
Meaningfulness	In different cultural societies, factors that give life meaning are often found in spiritual and religious beliefs [32]. Most countries in Southeast Asia are multicultural with many minority groups, resulting in a variety of religions [30]; the most commonly practiced religion is Buddhism, whose basic principles are often familiar to people of other religions.	Religious and spiritual beliefs
Participation	Participation depends on a balance between opportunities and limitations [14]. Associated with balance is the ability to participate and play a role in ordinary family and community activities [33]. Southeast Asian countries often have a high inter-generational co-residence, where children take care of their parents [27]. The Southeast Asian elderly participate mainly by giving advice to family and community members, mainly on health-related issues. Their accumulated life wisdom and spiritual capacities make this advice highly appreciated [33].	Family structure, role of elders, in-laws, siblings
		Being able to participate and having a role in usual community activities
		Being able to participate and having a role in ordinary family activities (bringing in money, food, cooking, cleaning)
Daily functioning	Contributing to a healthy feeling is the ability to be functional at physical, social, and economic levels. Being able to carry out daily responsibilities and activities enables a person to manage his/her life with some degree of independence [14]. Especially for Southeast Asian people, this has a positive effect on one’s perception of health [33,45]. A study by Nilsson et al. (2005) indicated ‘having the strength and physical ability to work’ is essential to be functional in daily life [33]. Mentioned as a determiner of this is having good health, which in turn is related to food. Therefore, the availability of sufficient and healthy food is considered essential for everyday functionality [33].	Availability of/barriers to healthy food
		Current/past work related activities
Quality of life	The WHO (1993) defines quality of life (QoL) as “an individual’s perception of their position in life in the context of the culture and value systems in which they live and in relation to their goals, expectations, standards and concerns” [45]. The WHO explicitly identifies culture as one of the important factors influencing QoL. Interpretations and expectations regarding QoL are likely to vary during a person’s lifetime and depend on their generation [33]; health, illness, or disability can also have an effect. According to Huber, one aspect of QoL is happiness. Uschida et al. (2004) indicated that substantial variations exist in the cultural meaning of happiness: personal achievement is its main determiner in the cultures of Europe and North America, whereas in Southeast Asia, it is defined mainly in terms of interpersonal connectedness [34,35].	Social network discussed, role of social structures in health, e.g., governmental and non-governmental organisations

## Appendix B

**Table A2:** Findings from stakeholder meetings and expert review meetings regarding facilitators and barriers of the guideline, and revisions made based on these findings.

Findings	Stakeholder Meetings	Global Expert Review	Quotes
Facilitators	Providing insight into the culture and context of end-users: especially helpful when implementing an intervention in another area or with other end-users. Guideline helps to adapt a medical message to the appropriate context.	Guideline should become routine when developing, implementing, or revising interventions. A tool that helps to deliver information in a culturally adapted way.	Participant from Indonesia: “*Some medical words were used in the materials that health practitioners use. However, not all community members know these words. Based on the checklist, we found this and revised this.*”
	Contextual aspects covered in the checklist are broader than merely health-related. Thus, the guideline can be applied beyond health-related CBHIs. Conceptual framework provides a broad perspective on health, including social and environmental aspects.	Guideline covers interventions beyond health, such as interventions to reduce natural disasters.	Participant from The Philippines: “*The guideline can be used more broadly, not only for health focus or topics. For example, for disaster reduction. It should also be culturally and contextually sensitive. Often we use or refer to materials from other countries, and we forget that we have a different context and a different culture.*”
Barriers	Several contextual aspects vary within a country, an area, and different groups. Implication: guideline needs to be customized: different contextual aspects can be important in different contexts.	To overcome this barrier, involve stakeholders in the early stage of development or revision. People from the community itself know what fits their culture best.	Participant from Indonesia: “*The aspects of religion and beliefs are not applicable in our country because this is a private or a sensitive issue. Therefore in every context, different contextual aspects are more or less important. This should be more clear.*”
	Language can be a barrier for global use; translation is a difficult process, and meanings can be lost.	For translation, deploy stakeholders, expert translators, or a combination. A combination is preferred: a higher quality of professional translation combined with community stakeholder translation.	Participant from Vietnam: “*It would be ideal to involve people from other stakeholders or the communities where the training will be implemented. We often write proposals to international NGOs and include material development and communication materials. When representatives from different groups are involved, we could have really powerful input from communities about what would be important for them for developing this training. I think it even goes back to proposal development, because this takes budget to do it well.*”
	Time-allocation: applying the guideline takes time; this could limit use of the guideline.	Allocate the budget for applying the guideline when writing a proposal for the development of interventions. Moreover, involvement of stakeholders will save time.	Participant from Vietnam: “*The guideline is developed in English. So we are faced with the language barrier when using it in different countries with different languages.*”
		Revise model of healthy lifestyle. Implementers are not always role models; this should be carefully considered.	Participant from Cambodia: “*It is too sensitive that an implementer or a trainer should be a role model for a healthy lifestyle. Someone can still deliver this message of healthy lifestyle and be overweight.*”
Revisions made based on barriers	Application of the guideline should be a continuous process; if changes to an intervention take place, e.g., different target group or other area, the guideline should be used as a tool for monitoring. The guideline should be seen as an aid and not mandatory.	Make clear that it is a tool, and not every aspect is applicable. If an aspect is not applicable, it can be ignored. In addition, make it clear that if topics or aspects are needed, they can be added to the checklist.	Participant from Vietnam: “*When implementing a training in a rural area and then in an urban area, there are differences between the target groups. With the guideline, we revised the training to make it more suitable for the local context. This can also be helpful when a training is adopted in another country.*”
	In contexts where social desirability and respect are highly valued, the implementer could feel restraint in writing down outcomes of the checklist. It could feel safer to discuss outcomes informally.	Open feedback questions can be added to the guideline. These questions can be put to the end-users to create discussion. Moreover, adding these questions involves stakeholders.	
	Add a textbox for lessons learned to contribute to transparency and suggest tips and tricks where needed.	Add suggestions as to who could be an observer. If no independent person is available, give options.	Participant from Vietnam: “*From past experience, I know how difficult it is to find observers. So maybe the suggestion could include options for the observers. If there are two or three trainers, that is not ideal, but they could take turns being the observer, and then give feedback together.*”
	Gender differences should be included in the checklist.	Ageism, disability discrimination, digital in-/exclusion and teaching methodology should be in the checklist. Cultural aspects of the visuals, such as posters, should also be in included.	
	Add hints and tricks on how to to develop trust and a feeling of safety among participants; this is important for cultural and contextual adaptation.	Explain that there are different levels of awareness of context in the process of adapting: organisational level, community level, and individual level.	Participant from The Philippines: “*There are different levels of awareness, at the organisational level, the individual staff level, the program level, or the organisational level. I think it is important to be aware of that and make it explicit.*”
