# Supplementary material for: A Guideline for Contextual Adaptation of Community-Based Health Interventions
Source: Int J Environ Res Public Health. 2022 May 10;19(10):5790. doi: 10.3390/ijerph19105790 (PMC9141251; doi:10.3390/ijerph19105790)
Supplement: Supplementary file 1 [file ijerph-19-05790-s001.zip › ijerph-1671174-supplementary.pdf]

Scaling-Up NCD interventions in South-East Asia

Guideline for adaptation of community-based health interventions to culture and context

This guideline is for developers and implementers of community-based health interventions, programs, trainings and materials

This project has received funding from the European Union's Horizon 2020 research and innovation programme under grant agreement No 825026.

Acknowledgements

This guideline is developed within the European Union's Horizon 2020 research and innovation programme of the Global Alliance for Chronic Diseases (GACD) - Scaling-up of evidence-based health interventions at population level for the prevention and management of hypertension and/or diabetes in Southeast Asia (SUNISEA). The funding source was not involved in the development of the guideline.

The development of the guideline was made possible thanks to the support and advice of many individuals and organisations. The guideline is established in collaboration with policymakers, researchers, experts, community workers and volunteers. The overall goal was the development of a guideline for intervention, training or program developers and performers to realize cultural and contextual sensitivity of their product. Organisations that were involved in the development are University Medical Center Groningen, HelpAge International, University of Public Health Myanmar, Thai Nguyen University, Health Strategy and Policy Institute Vietnam and Sebelas Maret University Indonesia. The review team comprised the SUNISEA Consortium members and HelpAge global experts.

Copyright ©2022

First Edition

Any parts of this publication may be reproduced for non-profit purposes unless indicated otherwise. Please clearly credit the SUNISEA Consortium and send a copy of the reprinted article or web link to: z.e.pardoel@umcg.nl

Contents

Acknowledgements 1
Contents 2
Introduction 3
1. Background 4
2. Why, who and how? 5
  Why adapt community-based health interventions to the local context? 5
  For whom is the guideline? 6
  How to use this guideline? 6
3. Phases of adaptation 7
  Phase 1: Preparation 7
    Step 1.1 Feasibility and need check 7
    Step 1.2 Identify and include resources and skills 7
    Step 1.3 Plan ahead and think of the steps after assessment 7
  Phase 2: Assessment 8
    Step 2.1 Checklist for cultural and social sensitivity of written materials 8
    Step 2.2 Checklist for cultural and contextual sensitivity of implementation 11
  Phase 3: Adaptation 13
    Step 3.1 Revisions 13
    Step 3.2 Follow-up 13
Appendix 1: Definitions and meaning of items 14

Introduction

This guideline is developed with the aim to guide the process of adapting a community-based health intervention, training or program to the local context. In the following text, the word intervention is used for intervention, training or program.
This guideline consists of the following chapters: The first chapter describes the *background* of this guideline. In the second chapter it is explained for who the guideline is and how it can be used. In the third chapter you will find the concrete steps you need to take to adapt your intervention, training or program.

The process of adapting a community-based health intervention consists of three phases: (1) the preparation phase, (2) the assessment phase and (3) the adaption phase. In the preparation phase practical information on what preparations are needed or recommended are described (see figure 1).
In the Assessment phase, we explain how to assess the materials and implementation.
In the last phase, the Adaption phase, a description of how to adapt the materials and/or implementation is given.

Figure 1 Illustration of the three phases of the adapting process

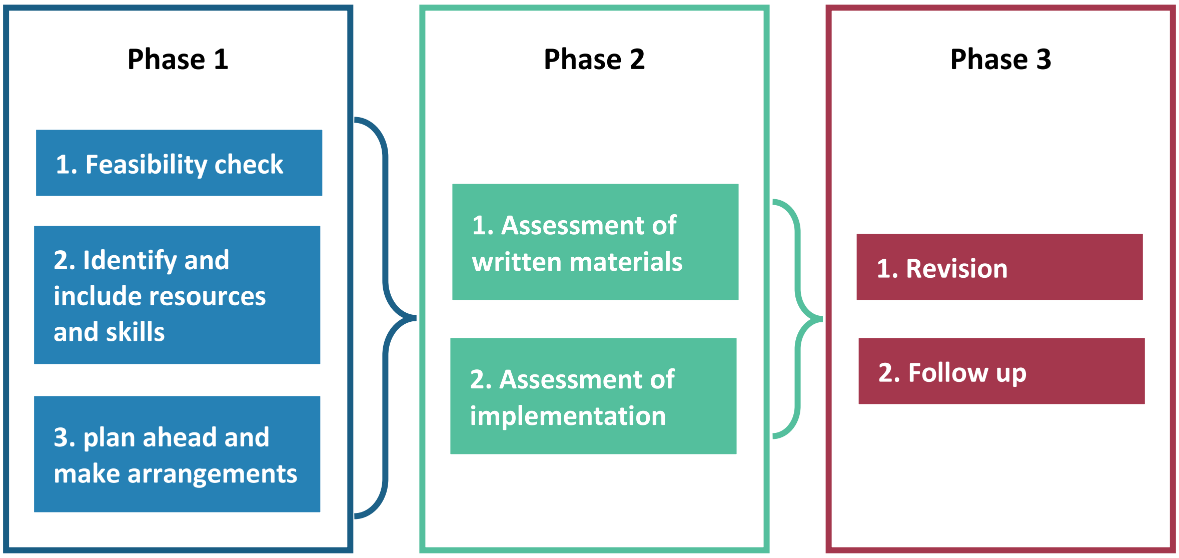

1. Background

Community-based health interventions (CBHIs) have been shown to promote health more effective when tailored to sociocultural specific aspects, for instance the local language, including the lingo, custom traditions and adapted to preferences, needs, values, interests and religion[1,2,3]. Therefor It is very important that intervention materials are adapted to local culture and that the implementers are culturally competent, taking local customs into account, address local taboos, and use appropriate instruments for interactive intervention.

CBHIs can be defined as: complex social processes, that include multi-component interventions, aimed at preventing illness and unhealthy behaviour and promoting well-being among population groups in a defined local community in their context[4]. Within these complex social processes, health perception is divergent. According to the 'Concept of Positive Health'[5,6] health perception can be divided in six major dimensions: *bodily functions*, *mental well-being*, *meaningfulness*, *participation*, *daily functioning*, and *quality of life*. These dimensions address health in a dynamic and comprehensive way, including multiple components and explaining that health is, next to physical functioning, influenced and affected by a person's social environment, real-life situation, ability to participate and meaningfulness. Health is established in and influenced by a persons' cultural, social, healthcare and demographic context.

---

[1] Gyawali B, Bloch J, Vaidya A, et al. Community-based interventions for prevention of Type 2 diabetes in low- and middle-income countries: a systematic review. Health Promot Int. 2019;34:1218–1230.

[2] Ku GM V, Kegels G. Adapting chronic care models for diabetes care delivery in low-and-middle-income countries: A review. World J Diabetes. 2015;6:566–575.

[3] Niazi AK, Kalra S. Patient centred care in diabetology: an Islamic perspective from South Asia. J Diabetes Metab Disord. 2012;11:30.

[4] Pardoel ZE, Reijneveld SA, Lensink R, et al. Core health-components, contextual factors and program elements of community-based interventions in Southeast Asia – a realist synthesis regarding hypertension and diabetes. BMC Public Health [Internet]. 2021;21:1917. Available from: https://doi.org/10.1186/s12889-021-11244-3.

[5] Huber M, Knottnerus JA, Green L, et al. How should we define health? BMJ. 2011;343:d4163.

[6] Institute for Positive Health. Positieve gezondheid. https://www.iph.nl/.

2. Why, who and how?

Why adapt community-based health interventions to the local context?

People have different perceptions of health, and give different weight to elements of health (body, mind, daily functioning, participation in society, quality of life, meaning of life). These differences can be based on individual preferences and ideas but can also have cultural and contextual components. By taking these cultural and contextual components into account, interventions and materials are more relatable to people's perceptions and more acceptable for them to use. If well aligned to people's health perceptions, the impact on health behaviour may be much higher, because people understand the key messages better.

Examples of cultural and contextual components that need to be taken into account are (see figure 3):

- Spirituality and religion
- Living in an urban or a rural setting
- High or low socioeconomic conditions
- Demographics, like age and gender
- Present stigma
- (Health) literacy/language
- Diet habits

Figure 2 Illustration of dimensions of Positive health influencing health perception with cultural and contextual aspects

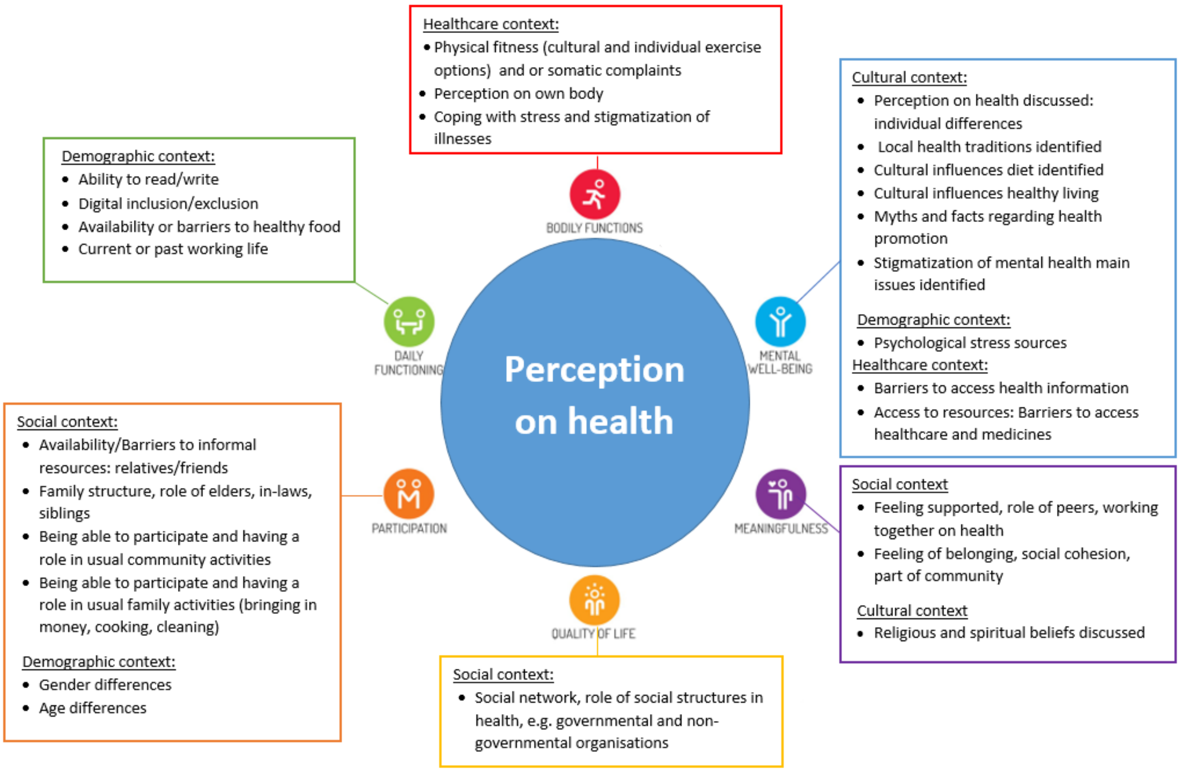

For whom is the guideline?

The guideline and checklists are developed to adapt CBHIs and materials to culture and context. It can be used by any person that is involved in the development, performance or adjustment of CBHIs and materials that target disease, health promotion or screening. The guideline is a tool, to support developers and implementers in cultural and contextual adaption of these CBHIs and materials.

How to use this guideline?

This practical and science-based guideline is a tool that helps the development or implementation of CBHIs. In this guideline, background information, instructions with tips and tricks on how to use the checklists, two checklists (one for development of materials and one for execution) and a list with definitions and meanings, are described. Also, it is explained when this guideline can be applied. Be aware that different contexts have different contextual aspects. Therefore, the importance and existence of aspects differ by context, and therefore not every aspect is relevant for your intervention. The guideline is not bound within CBHIs, it could be applicable to other interventions and settings as well.

In the next chapter you will find the three phased of adaptation explained and all the practical information you need to know to apply the guideline for cultural and contextual adaptation to your own intervention.

Please note that in this guideline, the following denotations are used: the developer and the implementer. The developer is the person who is involved in the development of an intervention or materials. The implementer is the person that actually implements the intervention.

3. Phases of adaptation

Phase 1: Preparation

In this phase steps to prepare the adaptation process are described.

Step 1.1 Feasibility and need check

The first step is to assess if cultural and contextual adaptation of the intervention is feasible and needed. There can be several reasons why assessing cultural and contextual adaptation can be needed, for example:

- Developing a new intervention
- An existing intervention is being implemented with a different target group
- An existing intervention is being implemented in another area/province/country
- An intervention was developed +/- 2 years or more ago
- Adjustments to the materials, such as adding new material to an intervention
- Any changes to the implementation, such as via a different implementation channel, for example from live meetings to virtual meetings

These are some examples of situations where it is feasible to adapt a CBHIs to the context. In this step it is important to formulate a clear reason for adaptation. Formulating a clear reason for adaptation can best done with the following criteria: Specific (simple, sensible, significant), Measurable (meaningful, motivating), Achievable (agreed, attainable), Relevant (reasonable, realistic and resourced), and Timely (time-based, time limited) (SMART)[7].

Step 1.2 Identify and include resources and skills

To preform cultural and contextual adaptation, the resources and skills should be available for assessment. For resources and skills, you can think of the person who is going to assess the intervention with the checklists: does he/she have the time and skills to do so. Moreover, can the materials be adjusted? Is there budget and time available?

Step 1.3 Plan ahead and think of the steps after assessment

Discuss and write down the plan for after the assessment of the intervention. In the plan write down how the materials and interventions are going to be adapted, for example who is going to be involved, who is going to adapt the intervention, will the assessment be done with pen and paper or electronically. Write down an estimation of the time needed for both checklists and for the adaptation.

[7] https://www.mindtools.com/pages/article/smart-goals.htm

Tips & tricks

- When in the process of writing a proposal for development of an intervention or training, then take the budget into account, time, skills and resources needed for assessing if cultural and contextual adaptation is needed and feasible and adjusting the materials. Moreover, think of involvement of stakeholders such as end-users, community members, community representatives or government- and funding agencies.
- Think about if translation of the guideline/checklists is needed, and if so think about who (expert or non-expert) will translate. For translation, stakeholders can be involved. A combination between an expert translator and a local stakeholder translator is recommended.
- Involve end-users/stakeholders/community members in an early stage. This helps the process of contextual adaption and can overcome practical issues, such as translation.

Phase 2: Assessment

After completing phase 1, the assessment phase can start. This phase includes using two checklists that are developed based on the concept of Positive Health and culturally sensitive care. The first checklist is for review of written materials. The second checklist is for review of the implementation of an intervention. In the checklists, per aspect remarks, i.e. any argument for change or notable findings, can be written down. This is important for the process, especially when there is collaboration between different stakeholders. Writing down the findings in the remarks box contributes to a transparent process and other stakeholders can understand the needed adjustments better. In the last row, lessons learned can be written down. In this row, overall potential adjustments you envision can be written down.

Note: not all domains and/or items are of interest or suitable in every context. Important to take the relevant domains and items into account and ignore the non-relevant domains and items.

Step 2.1 Checklist for cultural and social sensitivity of written materials

This step describes how to check the written materials for cultural and contextual sensitivity. This can be done before, during and after the development of materials. When the checklist is used before the development, it can be used as a preparation tool to ensure certain aspects will be covered. When using the checklist after development make sure you can still adjust the materials where needed.

For what and for whom

This checklist serves as an overall evaluation of written materials: Is the material culturally sensitive and adapted to the context? Developers of an intervention can use the checklist to verify the cultural and contextual aspects of an intervention. The checklist can be used by developers of an intervention or independent observers, or both. An independent observer is somebody who is not involved directly in the development of an intervention and has no direct benefits or disadvantage by observing it. A combination of developers and independent observers is preferred, because it contributes to the transparency of the process.

How to use the checklist

The checklist consists of eight domains, namely: general, bodily functions, mental well-being, meaningfulness, participation, daily functioning, quality of life and lessons learned. Every domain consists of one or more cultural and/or contextual items. The checklist can be used the following way:

- All items can be checked by a 'yes' or 'no'.
- A 'yes' means that the aspect is covered in the materials and a 'no' means that is not covered.
- If an item is not covered in the materials, the next step is to explore if the item is relevant for the intervention. For example, gender differences: if the participants are both female and male, this can be an important aspect to address. If the participants are only female, the aspect is not of importance.
- If the item is not of importance, an explanation for this should be written down in the remarks section.
- If an item is of importance, this means the materials should be adjusted.
- Based on all the checked items, the materials can be adjusted. In the next phase, the adaption process is explained.

Tips & tricks

- When an intervention, training or program will be implemented in another area, region, community, country or with another target group, it is recommended to review the materials with the checklist again.
- The review of the materials can best be done by at least two developers/observers, to ensure careful checking to determine accuracy. Make sure that consensus is reached.

Checklist for materials

Domain	Contextual/cultural aspects [8]	Yes	No	Remarks
**1. General**	1a. Gender differences of end users addressed			
	1b. Ability to read/write of end users addressed			
	1c. Materials adapted to age of end-users			
	1d. Visuals, e.g. pictures, posters, adapted to culture and context			
	1e.digital inclusion/exclusion			
**2. Bodily functions**	2a. Physical fitness (cultural and individual exercise options) addressed			
	2b. Somatic complaints addressed			
	2c. Perception of physical functions described (i.e. how does the community member see their own body functioning)			
	2d. Perception on health mentioned: individual differences explained			
	2e. Health traditions mentioned			
	2f. Cultural influences diet mentioned			
	2g. Cultural influences healthy living mentioned			
	2h. Disability status addressed			
**3. Mental well-being**	3a. Stigmatization of mental health mentioned			
	3b. Psychological stress mentioned			
	3c. Coping with stress addressed			
	3d. Stigmatization of illnesses addressed			
	3e. Availability/Barriers to informal resources: relatives/friends			
	3f. Access to resources: Barriers to access healthcare and medicines described			
	3g. Barriers to access health information mentioned (e.g. internet access, literacy). Include access to electronic media (internet, social media)			
**4. Meaningfulness**	4a. Religious and spiritual beliefs mentioned			
**5. Participation**	5a. Role of member in family/friends/community			
**6. Daily functioning**	6a. Current/past working life addressed			
	6b. Availability/barriers of healthy food addressed			
**7. Quality of life**	Covered in 3g and 5a.			
**8. Lessons learned or other remarks:**				

[8] For descriptions and meanings of the items see appendix 1

Step 2.2 Checklist for cultural and contextual sensitivity of implementation

This checklist is to evaluate/examine the implementation of the intervention. To what extent do the performers consider cultural sensitivity and contextual aspects? This checklist serves as a list to review the implementation of the intervention.

The checklist consists of nine domains, namely: general, bodily functions, mental well-being, meaningfulness, participation, daily functioning, quality of life, role of trainer/implementer and lessons learned. Every domain consists of one or more cultural and/or contextual items. Based on these items, cultural and contextual aspects of intervention implementation can be assessed.

The checklist should be applied by an observer. An observer is, preferably, an independent person who is not involved in the development or implementation of the intervention. The observer observes the implementation process and uses the checklist to review the incorporated cultural and contextual aspects. If there is no independent observer available, a trainer or participant in the intervention can fill in the checklist. The checklist should be used during the implementation of the intervention. This can be done during a pilot session or continuous.

Next to filling in the checklist, the observer can ask questions to the participants about the cultural or contextual aspects in the intervention. The observer should do this, to verify their own findings and/or gather new information. The findings from open questions should be written down on the checklist template. This can be in the remarks section, if it is about a certain aspect, or it can be written down in the lessons learned if it is about the intervention in general.

Tips & tricks

- The checklist can best be used during interactive activities between the implementer and the participants/attendees (in sessions where implementers and participants communicate back and forward).
- A way of using the checklist is involvement of participants. When feedback from participants is asked, (part of) the checklist can be incorporated in the discussion or during other ways of feedback. You could ask this using open questions, such as what did you like and what would you like to change?
- When an intervention will be implemented in another area, region, community, country or with another target group, it is recommended to review the implementation with the checklist again.
- When checking 8a, be aware that a healthy lifestyle differs for every continent/country/region/area/community/individual

Checklist for implementation

Topic	Contextual/cultural aspects[9]	Yes	No	Remarks
**1. General**	1a. Gender differences addressed			
	1b. Ability to read/write discussed			
	1c. Age friendly method used, addressing differences between generations. If end users are adults: Adult learning methods applied			
	1d. Digital inclusion/exclusion addressed			
**2. Bodily functions**	2a. Perception on own body discussed			
	2b. Physical fitness (cultural and individual exercise options) and or somatic complaints discussed			
	2c. Coping with stress and stigmatization of illnesses addressed			
**3. Mental well-being**	3a. Perception on health discussed: individual differences explained			
	3b. Local health traditions identified and discussed			
	3c. Cultural influences diet identified and discussed			
	3d. Cultural influences healthy living discussed			
	3e. Myths and facts regarding health promotion discussed			
	3f. Stigmatization of mental health main issues identified and discussed			
	3g. Psychological stress sources identified and discussed			
	3h. Barriers to access health information discussed			
	3i. Availability/Barriers to informal resources: relatives/friends			
	3j. Access to resources: Barriers to access healthcare and medicines discussed			
**4. Meaningfulness**	4a. Religious and spiritual beliefs discussed			
	4b. Feeling supported discussed, role of peers, working together on health			
	4c. Feeling of belonging discussed, social cohesion, part of community			
**5. Participation**	5a. Family structure discussed, role of elders, in-laws, siblings			
	5b. Being able to participate and having a role in usual community activities discussed			
	5c. Being able to participate and having a role in usual family activities discussed (bringing in money, food, cooking, cleaning)			
**6. Daily functioning**	6a. Availability/barriers of healthy food discussed			
	6b. Current/past working life discussed			
**7. Quality of life**	7a. Social network discussed, role of social structures in health, e.g. governmental and non-governmental organisations			
**8. Role of trainer/ implementer**	8a. Does the implementer represent or have knowledge about a healthy lifestyle			
	8b. Is the implementer a role model for target group			
	8c. Is the implementer culturally and linguistically matched to target group			
	8d. Participants treated equally and inclusively by the implementer			
	8e. No stigma or discrimination by the implementer, but inclusive and stimulating participants to come with solutions for local issues			
	8f. Does the implementer take cultural diversity of participants into account			
	8g. Does the implementer take different levels of knowledge of participants into account			
	8h. Is the intervention enhancing self-efficacy of participants			
**9. Lessons learned or other remarks:**				

[9] For descriptions and meanings of the items see appendix 1

Phase 3: Adaptation

After assessment of the intervention with the checklist, the materials and/or the implementation of the intervention can be adapted, based on the findings.

Step 3.1 Revisions

A first step in adaptation is reviewing the collected data. Are all necessary aspects checked? This can be done by reviewing the checklists. Moreover, check if the checked aspects are of relevance and can or should be revised, added or deleted in the materials and/or intervention.

When every aspect is checked, the lessons learned should be taken into account. In this section, information is shared about what has been learned from using the checklist and how you envision changes to the intervention. Based on this information, together with the checked aspects and remarks, the materials and/or implementation can be revised.

Tips & tricks

- For consistency, it is recommended to do the overall check with multiple persons. This could be a combination of another developer, an implementer, an observer or participant. Consult different stakeholders and ask for feedback.
- Keep a log (on paper or electronically) when revising materials or the implementation process. This way every revision and possible error can be traced. Moreover, it justifies the process of revision.

The revision of materials and/or implementation differs per intervention. It could be that written parts will be revised, added or deleted. It could be that the way of communicating, delivering a message, will change. All changes will eventually lead to the final materials and/or implementation. The final version(s) might be reviewed by different stakeholders to assess the applicability, consistency and quality of the revisions.

Step 3.2 Follow-up

To assure the applicability over time a follow-up of the checklist after an intervention is implemented, is recommended. The timeframe and content (e.g. assessing both checklists or only one, assessing all aspects or only certain aspects) for this depends on the intervention itself. Moreover, the follow-up can be done by consultation of involved stakeholders, such as the implementer or participants. This can be done by asking questions about their ideas and experiences regarding the contextual aspects.

Appendix 1: Definitions and meaning of items

Table 1 gives specific descriptions and meanings of the checked items.

Domain	Check developer or observer	Description*
**General**	Gender differences	The developer should check if materials are matched with gender of the target group, such as images and content. The implementer should address if the intervention is matching the gender of the target group. For example, a certain physical exercise can be less suitable for a gender. Health and health promotion can differ for gender. Moreover, health topics and interests can differ for gender. The implementer/developer should be aware of the gender differences.
	Ability to read/write discussed	The developer/implementer should check if participants are able to read and write to a certain extent that they can understand, follow and participate in an intervention.
	If end users are adults: Adult learning methods applied	The developer/implementer should apply learning methods appropriate for adults. Adult learning methods are:<br>1. Selective (learning what is meaningful for the participants),<br>2. Self-directed (participants choose how to learn, this can be visual, aural, printed materials, this differs per person. The developer/implementer should find out what every attendee prefers and should be able to provide different ways)<br>3. Be prepared that the participants have not been in a schooling position for a long period. Let them get used to being trained.<br>4. The developer/implementer should be aware that the participants have knowledge and experience, which influences their thoughts.<br>5. The developer/implementer should be aware of adults often having a problem-centered approach to learning. Participants want to see immediately how the content is relevant to their situation.
	Age friendly method used, addressing differences between generations	An intervention should be adapted to the age of participants. For example for younger people, using a mobile phone is easy, for older people this can be challenging. If participants of an intervention are divers in age, the developer/implementer should address differences between generations.
	Digital inclusion/exclusion	An intervention and the materials should be adapted to level of digital inclusion or exclusion of the participants. If materials or the intervention itself are digital, the level of being able to use digital devices, having accessibility to it and having the access to internet should be addressed.

Domain	Check developer or observer	Description*
**Bodily functions**	Physical fitness (cultural and individual exercise options) and/or somatic complaints discussed	The developer/implementer understands that the participants diverse in physical fitness and makes sure that participants have different/individual exercise options. Also, somatic problems (i.e. physical pain in body, injuries), which affect the physical fitness of participants are known and seen by the developer/implementer
	Perception of body discussed	The developer/implementer addresses the perception of participants their own body i.e. how does the participant see t own body functioning, satisfied or nor not.
	Coping with stress and stigmatization of illnesses addressed	A developer/implementer should address if and how participants handle stress and/or stigmatization of illness.
**Mental well-being**	Perception on health discussed: individual differences explained	Participants can have different perceptions on health. The developer/implementer should address if and what the perceptions on health of the participants are. If the differences occur, the developer/implementer should try to explain these differences.
	Local health traditions identified and discussed	The developer/implementer addresses what local health traditions participants have, i.e. particular health beliefs and traditions, like traditional healing, and taking of pills or injections.
	Cultural influences diet identified and discussed	The developer/implementer addresses if and what cultural influences on diet participants have, i.e. beliefs that certain foods are good for health, eating of certain products allowed or not.
	Cultural influences healthy living discussed	The developer/implementer addresses if there are cultural influences on healthy living among participants, i.e. cultural aspects that influence life, e.g. beetle nut-chewing, acceptance of smoking, eating transformed foods/sugary beverages associated with social class.
	Myths and facts regarding health promotion discussed	The developer/implementer addresses certain beliefs, myths or customs on health promotion among participants. This can be done by collecting and discussing a list of beliefs/myths/customs of participants.
	Stigmatization of mental health main issues identified and discussed	The developer/implementer addresses how participants see mental health, if there is stigmatization (such as religious beliefs in the devil, or mental health remains within the family.
	Psychological stress sources identified and discussed	The developer/implementer addresses sources of psychological stress of participants and discusses its impact/effects on participants.
**Meaningfulness**	Barriers to access health information discussed	The developer/implementer addresses if and what barriers to health information access are e.g. internet access, literacy. Include access to electronic media, e.g. internet, social media
	Feeling supported discussed, role of peers	The developer/implementer addresses if and how the participants feel supported and how they see the role of peers in health, i.e. do they work together with peers on health.
	Religious and spiritual beliefs discussed	The developer/implementer addresses certain religious and spiritual beliefs e.g. rituals, role of religious leaders, interrelation between religion and vaccination
	Feeling of belonging discussed, social cohesion, part of community	The developer/implementer addresses the extent of connectedness and solidarity among a community, friend group or family. In addition, the relationships that a person has with his family and/or friends and/or community. Do the attendees feel like he or she belongs to a community.

Domain	Check developer or observer	Description*
**Meaningfulness (cont.)**	Availability/Barriers to informal resources: relatives/friends	The developer/implementer addresses to what extent participants have informal resources such as relatives and friends and what the barriers to informal recourses are.
	Access to resources: Barriers to access healthcare and medicines discussed	The developer/implementer addresses access to healthcare resources, i.e. distance, transport, financial constraints.
**Participation**	Family structure discussed	The developer/implementers address the family structure of participants, i.e. roles of elders, in-laws, siblings, gender ro[illegible]
	Being able to participate and having a role in usual family activities discussed	Usual family activities could be bringing in money, food, cooking, cleaning.
	Being able to participate and having a role in usual community activities discussed	The developer/implementer should address if a participant is able to participate in community activities. For example d[illegible] the attendee have cultural beliefs that result in the attendee not wanting to participate in certain activities?
**Daily functioning**	Availability/barriers of healthy food discussed	The developer/implementer addresses the availability and possible barriers to healthy food of participants, e.g. costs of food, basic cooking utensils/lack of resources to cook "elaborated meals" increasing availability of processed and fast food, food preferences.
	Current/past working life discussed	Current or past working life is an important topic for how functional individual's feels. The implementer can address this by asking participants about their job-status.
**Quality of life**	Social network discussed, role of social structures in health	The developer/implementer addresses the social network of the participants and the role of social structures in health, [illegible] governmental and non-governmental organisations.
**Role of implementer**	Does the implementer represent/have the knowledge of a healthy lifestyle	The implementer is a person whose (health) behaviour and appearance is an example for the participants groups health knows how to live healthy
	Is the implementer a role model for target group	The implementer is a person whose behaviour, appearance, successes match with the participants.
	Is the implementer culturally and linguistically matched to target group	The implementer wears appropriate clothing, respects cultural norms of the intervention location, adapts to local conditions,<br>uses the same language (linguistic level) and appropriate words, and suits the age/culture/religion of the participants.
	Participants treated equally and inclusively by the implementer	The implementer treats all participants the same way, without excluding any member for any reason.
	No stigma or discrimination by the implementer, but inclusive and stimulating participants to come with solutions for local issues	The implementer does not label participants. The implementer does not shame or brand a person in any way.<br>The implementer includes all participants in all activities and stimulates participants to come with solutions for local iss[illegible]
	Is the intervention enhancing self-efficacy of participants	The implementer enhances member's belief in own capacity to execute behaviour. The implementer stimulates confidence of<br>the participants about their own motivation, behaviour and social environment.
	Does the implementer take cultural diversity of participants into account	The implementer takes cultural diversity of participants into account, i.e. culturally based activities, reflects on culture diversity.
